# Diet: A Specific Part of the Western Lifestyle Pack in the Asthma Epidemic

**DOI:** 10.3390/jcm9072063

**Published:** 2020-07-01

**Authors:** Carmen Frontela-Saseta, Carlos A. González-Bermúdez, Luis García-Marcos

**Affiliations:** 1 Department of Food Science and Nutrition, Faculty of Veterinary Sciences, Regional Campus of International Excellence “Campus Mare Nostrum”, 30100 Murcia, Spain; 2 Biomedical Research Institute of Murcia (IMIB-Arrixaca), University of Murcia, 30003 Murcia, Spain; cagb1@um.es (C.A.G.-B.); lgmarcos@um.es (L.G.-M.)

**Keywords:** Western lifestyle, saturated fats, simple carbohydrates, obesity, adipose tissue, inflammation, asthma

## Abstract

The Western lifestyle is a complex concept that includes the diet as the main axis of different factors which contribute to a detrimental effect on health, lower life expectancy and low quality-of-life. This type of diet is characterized by being high in calories, mainly provided by saturated fats, and rich in sugars that can lead to changes in immune cells and their responsiveness, by different mechanisms that have yet to be totally clarified. Inflammatory processes are perpetuated through different pathways, in which adipose tissue is a major factor. High fat stores in overweight and obesity accumulate energy but the endocrine function is also producing and releasing different bioactive compounds, adipokines, known to be pro-inflammatory and which play an important role in the pathogenesis of asthma. This review therefore explores the latest evidence regarding the adverse effect of the Western diet on adipose tissue inflammation and its causative effect on the asthma epidemic.

## 1. Introduction

Inflammatory diseases are increasing worldwide to epidemic proportions and are considered lifestyle-associated diseases, similar to obesity [1]. These pathologies have a multifactorial cause in which diet is a well-known environmental factor involved in obesity, whilst in the etiology of other inflammatory diseases, diet is gaining increasing attention as a risk factor. With regard to this, the Western diet is characterized, broadly speaking, by a high content of saturated fats and simple sugars and a low content of plant-origin foods, and is associated with an increased risk of inflammatory diseases, including asthma.

This kind of diet usually includes foods rich in calories and their regular consumption can lead to overweight and obesity, which basically consists of an excess of body-fat stores. This adipose tissue seems to be an important factor in systemic inflammation, including airway inflammation (asthma), and especially in the case of obesity, which is characterized by excessive accumulation of fat [2]. From this perspective, this review examines the evidence for the association between asthma and nutrition, and specifically addresses the effect of Western diets on the inflammatory processes, reviewing the causative relationships with the adipose tissue.

## 2. Diet and the Parallel Epidemics of Obesity and Asthma

There is a growing body of evidence on the association between asthma and nutrition. Although the exact mechanisms of this association are far from clear, the epidemiological data suggest that this is indeed the case.

From the most elemental point of view, there has been a parallel increase between the asthma and the obesity epidemics [3], with the latter being closely related to changes in nutrition [4]. According to the epidemiological data on asthma tracking back to the 1960s and 1970s [5,6,7], in the Nordic and English-speaking countries, there has been an increase in asthma prevalence in all age groups. That same trend has been found in Mediterranean countries [8]. Those regional reports were later supported, albeit partially, by the findings from the International Study of Asthma and Allergies in Childhood (ISAAC), which compared prevalence data from its Phases One (carried out between 1994 and 1995) and Three (performed between 2002 and 2003) [9]. There was, however, a different note here as the authors suggested that the prevalence had increased among both schoolchildren as well as adolescents in those countries where the asthma prevalence had previously been lower, while it was stable or even reduced in countries with a formerly higher prevalence.

The obesity epidemic, which relates to nutrition and sedentarism, tracks back to similar years to that of asthma and shows a parallel increasing trend starting at a higher prevalence [3]. This does not necessarily mean that the two epidemics really started at the same time, but the epidemiological data available track back in an interesting parallel way. Curiously, a similar behavior to the asthma epidemic seems to be happening at least in some populations: both in adults and adolescents, the rise in prevalence occurring from the 1970s seems to have slowed down or even halted [10,11].

Taking this phenomenon into consideration, whether nutrition disorders lead to obesity and this to asthma (through altering lung mechanics, for instance), or asthma favors obesity (through sedentarism, for example), or asthma and obesity are effects of common parallel causes (including nutrition) is a matter of certain debate and is probably dependent upon each individual.

There are certain facts that relate obesity to asthma, starting from a common genetic predisposition, as shown by studies in twins. The study by Hallstrand et al., including 1001 monozygotic and 383 dizygotic twin pairs of the same sex, arrived at the conclusion that apart from there being a strong association between asthma and body mass index (BMI), there was also an important heritability for asthma (53%) and obesity (77%) with additive genetic influences in each condition [12]. However, a second study from the Danish Twin Registry [13], including 29,183 twin individuals, found that the heritability of obesity was higher (81% in males and 92% in females) as was that of asthma (78% and 68%, respectively). However, their analyses of age-adjusted genetic liabilities to asthma and obesity were significantly correlated only in females, and this was related to common genes.

Nevertheless, diet has been shown to be independently associated to asthma at the epidemiological level. For instance, in cross-sectional studies, a Mediterranean diet has been shown to be associated to a lower prevalence of asthma, independently of BMI [14,15], whilst frequent fast-food consumption seems to be a risk factor for asthma [16]. Furthermore, the frequent consumption of anti-oxidant foods has been associated to lower asthma prevalence [17,18]. Those dietary profiles (Mediterranean diet versus fast food) also have important implications for obesity. On the other hand, the frequent consumption (three or more times per week versus never or occasionally) of individual foods such as fruit and vegetables have been related to lower BMI and to lower asthma prevalence in adolescents worldwide [16,19].

More interestingly, the influence of certain factors related to both asthma and obesity seem to be important during pregnancy or during the first weeks of life. For instance, adherence to a Mediterranean diet by the mother in pregnancy seems to have implications for both asthma and obesity [20]. Moreover, maternal obesity in pregnancy is associated to both asthma and obesity in the offspring [21,22].

Thus, it is quite probable that nutrition influences obesity and asthma in parallel, independently of the interaction of the two conditions on each other, no matter whether the mechanisms of this interaction are purely mechanic, inflammatory, or others, and this influence occurs more easily in genetically predisposed individuals. The purpose of this review is to summarize the current evidence on the connections between nutrients highly present in the Western diet and asthma, as well as the possible mechanisms involved.

### 2.1. Role of Micronutrients and Macronutrients

The relationship between environmental factors, such as diet, and asthma risk is very complex and thus not fully understood [23]. The effects of individual nutrients on health have been widely studied in order to better understand the effects of isolated compounds on different diseases. However, nowadays, the study of specific dietary patterns and foods as complex matrices and their effect on health and disease prevention is gaining attention [24]. Regarding this, diets rich in vegetables are clearly related with valuable effects in preventing different diseases, whilst diets rich in calories, and overall when that energy is mainly provided by fat-rich foods, are strongly linked with chronic inflammatory processes, such as asthma, among others [25]. These fat-rich foods, usually consumed as part of the Western-style dietary patterns which are also considered as obesogenic, are often associated to an increased access to highly processed foods that are related with high contents of simple sugars and saturated fats and low contents of minerals and vitamins [26]. It is clear that this change in dietary patterns modifies general diet quality, which is involved in the development of different diseases, mainly those related with inflammation, cardiovascular risk, aging process [27], and also with the increasing burden of asthma [28], mainly through the control of various immune pathways; specifically, with the role of macro- and micro-nutrients on them being clearly different.

This type of pattern (Western diet) also includes reduced intake of micronutrients (vitamins and minerals), dietary fiber, unsaturated fatty acids and a low consumption of a wide variety of bioactive compounds that, in isolation, have been reported as having different interesting beneficial properties [29], and when they are consumed as part of a diet, may act together with more favorable effects on health status and disease management, including asthma [30]. Furthermore, typical Western foods are usually poor in fiber, providing daily amounts below the recommended 30 g, which is correlated with a higher risk of respiratory diseases related with an increase in short chain fatty acids’ (SCFA) production in the colon, which are systemically distributed [31].

Within the dietary foodstuffs included in the Mediterranean diet, fish consumption is highly recommended and implies the intake of fatty acids with a predominance of the w-3 profile that are able to partly inhibit a number of aspects of inflammation [32] and have been proposed as protective metabolites for asthma. However, this effect has been demonstrated when marine oil is abundantly consumed in the diet, provided by one serving of fish per day. This, although it can be obtained through the diet, is not a usual habit. Based on this, including 2–3 portions per week of sardines, mackerel, herring, tuna or salmon in addition to foods rich in different compounds with anti-inflammatory activity, such as those included in the Mediterranean diet, would help to achieve sufficient amounts of bioactive compounds to reduce inflammatory pathways.

The increased distance from this dietary pattern has alarmingly increased overweight and obesity, as well as related diseases, in recent years [33]. In obesity, excessive weight gain leads to adipose tissue remodeling, adipocyte hypertrophy, hypoxia, stress and apoptosis/necrosis, including a prolonged production of inflammatory mediators, with the subsequent release of adipose-derived pro-inflammatory cytokines and free fatty acids into the circulation. This can lead to systemic disturbances in metabolism and tissue health, promoting chronic low-grade inflammation and an increased risk for chronic diseases [34]. With regard to this, it has recently been reported that fat mass loss compared to BMI or weight can further improve different risk profiles and inflammation-related biomarkers and also shows a high capability for predicting the cardiometabolic profile [35]. This highlights the importance of body fat in the inflammatory response and how the accumulation of excessive fat, as in the case of obesity, may interfere with the maintenance of an optimal state of health [2]. Inflammatory mediators are stimulated by macronutrients in the adipose tissues that are mainly incorporated as flux through the diet as carbohydrates and fats that, after absorption, are stored in adipose tissue. It is important to highlight that not only the total amount of calories consumed but also their distribution, mainly across fats and/or carbohydrates, have a different impact on adipose tissue and thus on the incidence/severity of asthma [2,36].

Overconsumption of macronutrients in the diet stimulates the adipose tissue to release inflammatory mediators, predisposing the pro-inflammatory state in addition to increasing the risk and severity of infections [37,38]; moreover, micronutrient deficiencies (especially regarding those which are lipophilic: vitamins A, D, E, K and carotenoids) have a negative impact on the regulation of adipose tissue biology with respect to the modulation of adipogenesis or inflammatory status that gain importance related with obesity and associated pathologies. Regarding this, there is also information indicating that diet composition (the type of dietary macro- and micro-nutrients), independently of its caloric content, can influence the function of adipose tissue in different ways and the expression and secretion of inflammatory biomarkers [37].

In this way, complex dietary carbohydrates (starches, glucans, fructans and cellulose), and especially those from whole-grain products, have demonstrated an inverse association with inflammation and adipose tissue deposition but also depending on the gut microbiota population [38]. Meanwhile, elevated simple carbohydrates’ (sugars mainly used as sweeteners, such as glucose and fructose) consumption promotes the inflammatory state and acts on adipose tissue, inducing lipogenesis, because, in excess, they are converted into fatty acids, mainly palmitate, and promote lipid synthesis. Simple carbohydrates are highly consumed in the Western diet, and based on their effects on health, we can affirm that limiting the consumption of simple/refined grains and increasing the intake of whole grains is highly recommended [39]. Moreover, the high consumption of simple sugars used as sweeteners in different sugary beverages, that are widely consumed in this kind of diet, has been associated with asthma being more evident in case of beverages containing a high fructose:glucose ratio that can cause fructose malabsorption, resulting in the intestinal formation of pro-inflammatory products between unabsorbed fructose and some dietary proteins that, after intestinal absorption, are associated with asthma [40].

Focusing on dietary fats, they are mainly consumed as triglycerides and great differences and biological effects on tissues can be found, depending on the type of fatty acid after lipolysis. An increase in the intake of monounsaturated fatty acids (MUFAs) (mainly oleic acid, abundant in olive oil), omega-3 polyunsaturated fatty acids (PUFAs) (alpha linolenic acid, abundant in seeds and vegetables, and eicosapentanoic (EPA) and docosahexanoic (DHA) acids, present in fish), and omega-6 (linoleic acid) present in nuts and seeds, particularly as a replacement for saturated fats, have demonstrated beneficial effects on health and a reduction in disease risk and in the case of asthma exerting benefits mainly related with the development and resolution of airway inflammation [41]. Furthermore, the saturated fats, which are highly consumed as part of the Western diet, have pro-inflammatory abilities involved in asthma, amongst other detrimental effects on health. Moreover, the weight loss usually associated to dietary saturated fats’ restriction, and the subsequent reduction of adipose tissue, also contributes to a reduction of neutrophilic airway inflammation. This has been recently described in the postprandial period after the intake of foods rich in saturated fatty acids, and also describing that dietary fat is more pro-inflammatory than simple carbohydrates in the case of asthma [42].

It has been reported that individual macronutrients exert a different impact on adipose tissue and inflammatory response [34,43] so that simple sugars and saturated fats, individually and/or combined in the diet, have the ability to induce cytotoxicity and oxidative stress, favoring inflammatory processes and even epigenetically reprogramming the immune response to more severe diseases, as occurs when the Western diet is regularly consumed [44]. Evidences on the negative effect of an unhealthy diet on respiratory health are robust, with dietary components of this type of diet being suggested as pro-inflammatory, inducing to low-grade systemic inflammation and influencing asthma development and severity. Regarding this, studies on the use of the Dietary Inflammatory Index (DII) [45] to predict the anti-inflammatory capacity of the whole diet in the case of asthma are still scarce and warrant further investigation but, they indicate that DII is higher in subjects with asthma and also indicate worse clinical asthma outcomes, indicating that an improvement in this index as an indicator of an adequate diet might be a useful strategy for improving clinical outcomes in asthma [45].

### 2.2. Role of Food Groups and Dietary Patterns

In this context, the Mediterranean diet reflects a complex concept which includes specific dietary patterns of a high consumption of olive oil as the main source of dietary fats, fruits, green vegetables, nuts, whole cereals, lean protein such as fish, moderate consumption of fermented dairy products and a limited use of meat, meat products and refined sugars [46]. This dietary pattern also includes high consumption of different compounds such as flavonoids, resveratrol or turmeric, among others [43], which are mainly present in vegetables, fruits, olive oil and nuts, and are very powerful against oxidative and inflammatory processes that are highly connected to pathways of the immune system. It is well known that a stable inclusion of this kind of diet, including a wide variety of foods, predominately of plant origin, provides solid health benefits in the prevention and also therapeutic approach of cardiovascular diseases, obesity, type 2 diabetes, metabolic syndrome, cancer and neurodegenerative diseases [47]. Antioxidant and anti-inflammatory properties of compounds present in foods consumed as part of the Mediterranean diet have also demonstrated effectiveness against inflammatory processes and it must be considered that moving away from these dietary patterns usually involves a high intake of processed foods rich in refined starches, sugar and saturated fatty acids that is often accompanied by a lower intake of fish, vegetables, fruits, nuts, legumes and whole grains. Antioxidants are molecules that scavenge free radicals, preventing oxidative damage. If the antioxidant defense system of lungs is unbalanced by oxidants, it can result in pulmonary dysfunction that could be buffered by dietary antioxidants present at high values in plant foods (vitamins C and E, carotene, flavonoids, selenium, etc.). These compounds have been shown to confer a protective effect on neutrophil membranes against oxidants exposure, improving immune cell function and contributing to the positive effect of plant foods’ consumption on asthma [48].

However, in the case of the Western diet, it is usually linked to a high consumption of processed foods and also supposes a high presence of saturated fatty acids in addition to a high intake of fat. This kind of dietary fat is strongly linked to inflammation in the adult stage. However, during infancy and childhood, high consumption of these saturated fats may also cause an activation of the innate immune system by excessive production of pro-inflammatory cytokines associated with a reduced production of anti-inflammatory cytokines [49]. This situation must be taken seriously because a gradual shift away from traditional diets to those higher in saturated fats, refined carbohydrates and animal-sourced foods, with increased processed food consumption and also changing culinary practices, has been confirmed [50]. Regarding this, the World Health Organization (WHO) [51] has established a global strategy on diet and health with the aim of reducing unhealthy diets and preventing different diseases, mainly those related to overweight and obesity, including asthma. This improvement of diets includes focusing on promoting fruits, vegetables, legumes, whole grains and nuts, and limiting saturated fats in favor of unsaturated fats, as well as promoting the consumption of foods rich in micronutrients that could reduce associated diseases and mortality.

Processed meats and red meats are also included in the Western dietary pattern and, interestingly, there are studies indicating that a high consumption of these meats has been positively associated with obesity [52]. Regarding this, despite the fact that consumption of cured meats, known for its high nitrite content, may favor airway inflammation and lung damage by nitrosative stress, few studies have been conducted on the association between processed meat and asthma. However, in the case of cured meats, that are an important component of the Western diet, a high intake of this kind of processed meat has been associated with worsening asthma symptoms, probably non-mediated by BMI [53,54], but is important to consider that this Westernized diet includes the consumption of complex meals that might interact with each other and that previous studies [55] have suggested that cured meat may adversely affect lung health, but the magnitude of the cured meat–asthma association may depend on other factors, including obesity or smoking, and must be considered within a broader context, also including dietary patterns and not only cured meat intake. Dietary patterns included in the Mediterranean diet involve a high consumption of fruits and vegetables containing antioxidant compounds that are capable of reducing nitrite levels with an anti-inflammatory effect in lung epithelial cells [55] and probably dampening the effects of high cured meat consumption on asthma symptoms. Moreover, epidemiological studies indicate an increased risk of inflammatory processes when a Mediterranean-style eating pattern is taken away, associated with a high consumption of processed meat, dietary saturated fats and low levels of vitamin D, that can reduce a tolerogenic mucosal immune state locally at the gut but also systemically, and particularly in the lung. In the case of vitamin D, that can be obtained from the diet or by dermal synthesis, its deficiency has been associated with a greater disease activity and extended disease duration in patients with different inflammatory processes, including protection against infections and regulatory effect on the gut microbiota, and has also been linked to beneficial effects in asthma [56].

## 3. Obesity-Related Asthma and Interrelations with Diet, Inflammation and Adipose Tissue

The parallel trend between the obesity epidemic and asthma makes it necessary to understand mechanisms involved in this association and how different components of foods included in the Western diet can be involved in the regulation of mechanisms in obesity-related asthma. In this regard, there are contradictory studies indicating that obese asthma is poorly controlled by conventional therapies, including corticosteroids, and it shows an increase in neutrophilic airway inflammation; however, there is a growing consensus on the important implication of fatty acids, inducing modifications on lipid metabolism and its immune regulators in obesity-related asthma [57,58]. Obesity has been linked to increased systemic leukotriene inflammation in patients with asthma and the excess of adipose tissue might contribute to airway inflammation, exacerbating asthma symptoms [59]. The inflammatory effect of obese asthma appears to occur through innate immune pathways, with a significant increase in the proportion of neutrophils in the airways of obese asthmatics [57]. In obese asthma, it is important to highlight that body composition and fat distribution affect systemic inflammation, airway inflammation and lung function, and this can explain important differences found between obese males and females with asthma, suggesting that the worsened lung function known to be associated with the obese-asthma phenotype is multifactorial and involves the body composition [60]. Western diet it is usually linked to a high consumption of foods containing important amounts of calories, mainly provided by fats and carbohydrates. In the case of fats, processed foods included in this type of diet provide high amounts of saturated fatty acids in addition to a high intake of fat and calories, increasing the risk of overweight and obesity, that contribute to immune dysfunction as well as altered airway structure and function [61]. The disturbed lipid metabolism and immune modulators of lipid metabolism in obesity, in addition to several immune factors, potentially contributing to the pathogenesis of obesity-related asthma, including intestinal microbiota and inflammation, could indicate that controlled modifications in the diet, in addition to a medical intervention, could be a promising strategy in controlling obesity-related asthma. These modifications in diet (mainly on fats and fiber types and contents) can also exert an important impact on human gut and its microbiome, leading to the selection of a high variety of bacteria interacting both for defense and nutritional advantages. Gut dysbacteriosis might result in altered immune response and chronic inflammatory respiratory disorders, particularly asthma (gut-lung axis), with an important role of the microbiome in inflammation and its influence on important risk factors for asthma being reported. Due to its high content in saturated fats and low fiber content, the Westernized diet can be a major contributor that can trigger factors regulating the development and/or progression of inflammatory conditions, including asthma. Increasing evidence indicates that there is a link between the gut and airways in disease development, reinforcing the evidence on the impact of the Western diet and associated nutrients on immune response and microbiota diversity, and how these can influence the pathology of asthma. Differences found on the lung microbiome between asthmatic and healthy people suggest that bacteria can contribute to the development of asthma, also indicating a possible important role in influencing the immune responses for gut microbiota [62].

Chronic inflammatory diseases are now considered epidemic and highly related with overweight and obesity. All these non-communicable diseases are considered a pandemic of lifestyle-associated pathologies [1]. As findings linking a chronic consumption of Western diet with inflammatory diseases such as asthma are consistent, efforts are now being focused on the study of how diets and combined components of foods can modify immune cell responsiveness. Different microbial metabolites produced after the digestion of foods seem to shed light on how immune cells are shaped in different ways depending on the type of diet. In the case of the Western diet—high in simple sugars and saturated fats—it can alter immune cell responsiveness, inducing systemic inflammation, which plays a key role in asthma patients.

Apart from lipid storage, other biological functions have been attributed to adipose tissue, such as hormones and protein factors’ production. These products are known as adipokines, playing different local and systemic roles with the main purpose of the integration of metabolism and immune systems. Pro-inflammatory cytokines such as leptin or resistin are included among them. In the same way, anti-inflammatory adipokines such as adiponectin have also been identified [63,64,65].

### 3.1. Anti-Inflammatory Adipokines: Adiponectin

Adiponectin is the best known and most abundant anti-inflammatory adipokine. It is secreted as a monomer, assembling and forming oligomers of different molecular weights. As a result, low, middle and high molecular weight (LMW, MMW and HMW) isoforms have been identified in serum [66] and their receptors, T-cadherin, adipoR1 and adipoR2, are widely distributed. The interaction between adiponectin and Adiponectin receptor 1 (AdipoR) receptors increases intracellular Adenosine monophosphate (AMP) concentration via AMP-activated protein kinase (AMPK) in different tissues and immune cells, with anti-inflammatory effects and AdipoR activation by HMW isoform, and seems capable of reducing tumor necrosis factor-alpha (TNF-α), transforming growth factor beta (TGF-β), interleukin-6 (IL-6) and interleukin-8 (IL-8) cytokines [67]. Adiponectin plasmatic concentration has been mainly associated with adipose tissue repletion, in the way that low calorie intake increases, whereas obesity decreases adiponectin levels [68,69,70].

Different studies have analyzed the relationship between serum levels of adiponectin and metabolic diseases. In this regard, Zhu et al. [69] and Iwata et al. [70] reported that a high HMW/total adiponectin ratio is positively associated to peripheric insulin resistance, whereas the association with other isoforms still seems to be unclear. According to the meta-analysis performed by Liu et al. [71], plasmatic levels of adiponectin can be considered as an adequate biomarker for prediction of metabolic syndrome risk. With regard to T-cadherin receptors, they have been related to a protective effect of adiponectin in heart and lung diseases [72]. In fact, different studies based on mice models pointed to the possibility that deficiencies in adiponectin and T-cadherin receptor could be related to myocardial ischemia-hypertrophy and high blood pressure [73,74,75].

At the bronchio-alveolar epithelium, T-cadherin seems to act as a binding protein, translocating adiponectin from serum to epithelial cells through the capillary barrier, playing an important role in inflammatory response regulation [74,76]. However, this mechanism still remains unclear. On the one hand, Otero et al. [77] proposed that adiponectin could have a pro-inflammatory effect, stimulating epithelial IL-8 secretion. Regarding this, Jaswal et al. [76] recently conducted a cross-sectional observational study comparing adiponectin levels between a group of 60 patients with chronic obstructive pulmonary disease, and 30 healthy people. That study concluded that a high level of adiponectin could have a pro-inflammatory effect, being positively correlated with airway inflammation and inflammatory biomarkers such as IL-8, whereas it was inversely correlated with forced expiratory volume and pulmonary function. On the other hand, Kirdar et al. [78] reported that the presence of a high level of adiponectin could be an attempt to reduce pro-inflammatory cytokines in chronic obstructive pulmonary disease, leading to a downregulation of TNF-α production by macrophages at the bronchio-alveolar epithelium. With regard to asthma, there are no conclusive results about the role of adiponectin on airway inflammation regulation in humans. It seems that serum adiponectin levels would be related to asthma severity, especially in children [79]. Ma et al. [80] analyzed serum adiponectin levels and BMI in 122 asthmatic children, concluding that asthma severity was negatively correlated with adiponectin levels, and positively correlated with BMI. These results are in concordance with previous studies showing that obese and asthmatic children presented an inadequate asthma control and lower serum levels of adiponectin when compared with non-obese asthmatic children [81,82]. The effect of low levels of adiponectin in obese-related asthma could be associated to an increase in TNF-α secretion by macrophages, favoring a Th2-predominant reaction and an eosinophilic-mediated inflammation (type I hyper-sensitivity) [74,82]. Recently, Zhu et al. [83] analyzed the effect of venous adiponectin administration in a murine model of obesity-related asthma, at a cellular and molecular level. According to the results they obtained, obese and asthmatic mice show low levels of serum adiponectin, as well as low levels of lung AdipoR1 and AdipoR2 receptors. Venous administration of adiponectin resulted in a reduction of eosinophils and pulmonary inflammation signs through apoptosis promotion of inflammatory cells, mainly mediated by two mechanisms: downregulation of the inhibitory apoptosis gene Bcl-2, and inhibition of TNF-α secretion. In the same way, administration of adiponectin increased activation of AMPK via AdipoR1 receptors, and inhibited nuclear factor kappa-light-chain-enhancer of activated B cells (NF-kB). Both the activation of AMPK and the inhibition of NF-kB were related to a significant reduction in nitric oxide (NO) species and inducible NO synthase (iNOS), as well as to a significant increment in total antioxidant capacity reported in serum and lung tissue from obese and asthmatic mice.

### 3.2. Pro-Inflammatory Adipokines: Leptin and Resistin

Leptin was the first adipokine described, being originally defined as a satiety hormone due to its effect on the satiety center in the hypothalamus. Its secretion is mainly regulated by adipocytes’ repletion, with its plasmatic levels positively correlating with adipose tissue. When the adipocytes’ lipid storage increases, leptin is released in order to stimulate anorexigenic peptides secretion by the hypothalamus [84,85]. However, a pro-inflammatory and immuno-modulatory effect has also been proposed [86,87]. Leptin is a 16 kDa non-glycosylated protein encoded by the *ob*-gene and it is recognized by cell receptors Leptin receptors (LEPRs), which belong to the type I cytokines superfamily. The leptin-LEPR complex activates different intracellular signaling pathways, mainly mediated by four tyrosine Janus Kinases (JAK_1_, JAK_2_, JAK_3_ and tyrosine kinase (TYK_2_)) and seven signal transducers and activators of transcription (STAT_1_–STAT_7_). Other intracellular pathways activated by leptin are the mitogen-activated protein kinase (MAPK) cascade, the phosphoinositide 3-kinase (PI3K) pathway or the 5′-AMP-activated protein kinase (AMPK) cascade [88]. LEPRs are expressed by the majority of immune cells, where the JAK_2_, STAT_3_, MAPK and PI3K pathways are activated in order to modulate both innate as well as adaptive immune response [44,89]. With regard to innate immune response, leptin promotes cell proliferation and decreases apoptosis, as well as stimulating the activity of NK-cells, activating chemotaxis and the oxidative function of neutrophils, activating the proliferation and pro-inflammatory cytokines release by eosinophils and basophils, or inducing proliferation, activation and pro-inflammatory cytokines production (such as IL-1, IL-2, IL-6, IL-8 or TNF-α) by monocytes, macrophages and dendritic cells. Among leptin’s effects on the adaptive immune response, the following have been described: induction of T cell proliferation, polarization to a pro-inflammatory TH_1_-IFNγ predominant phenotype instead of to an anti-inflammatory TH_2_-IL-4 phenotype, reduction of regulatory T cells and an increment of TH_17_ population favoring the maintenance of inflammatory and auto-immune response, proliferation and activation of B cells’ population [90].

Resistin is another adipocyte signaling molecule synthesized not only by adipose tissue, but also by monocytes and macrophages. Its secretion is induced by high glucose levels and inflammatory mediators (lipopolysaccharides, IL-6 or TNFα) [91]. Resistin has been mainly associated to insulin resistance in mice; however, in humans, it seems to actively regulate inflammation. This regulatory effect is mediated by adenylyl cyclase-associated protein-1 (CAP-1) receptors, presented in monocytes and macrophages. Resistin-CAP-1 complex increases intracellular AMP concentration and protein kinase A (PKA) activity, as well as activating DNA transcription regulated by nuclear transcription factor NF-kB. In this way, resistin upregulates the production of pro-inflammatory cytokines such as IL-6, IL-12 or TNFα [90,91].

Based on the above, leptin and resistin have been related to metabolic syndrome- and chronic inflammation-associated diseases. In this way, elevated circulating adipokines levels seem to be involved in the regulation of inflammation and allergic response, affecting the risk of asthma, especially in the case of obesity when adipose tissue is expanded and altered.

As previously explained, obesity has been associated to an increased risk of obstructive airway diseases and severe forms of asthma. In the same way, leptin serum levels are related to obesity and adipocytes’ repletion. For these reasons, different studies have tried to elucidate the possible role of leptin in airway inflammation and asthma. Regarding this, Sood et al. [92] performed a cross-sectional study which included 5876 participants (>20 years old), in order to measure basal morning serum levels of leptin, and to establish a possible correlation with clinical asthma status. The results obtained showed that high serum levels of leptin were associated with asthma, and that the association was stronger in women than in men. However, the relationship between BMI, leptin levels and asthma was not corroborated referring to the possible intervention of other factors. In the case of children, Guler et al. [93] also found a positive relationship between asthma and leptin serum levels. Bodini et al. [94] measured the level of leptin in serum and exhaled breath condensate (EBC) in 61 asthmatic and non-asthmatic children from 6 to 14 years old. Those authors also considered the obese and non-obese status based on the BMI. According to the obtained results, although no significant differences were found between obese and obese asthmatic children, they presented significantly higher levels of leptin in EBC and serum than non-obese asthmatic and non-asthmatic children. In the same way, leptin in EBC was significantly higher in non-obese asthmatic children than in non-obese and non-asthmatic children, without differences in serum levels of leptin. From this study, it can be deduced that leptin is able to translocate from serum to the respiratory epithelium, where it could play a crucial role in airway inflammation. This role seems to be more noticeable in non-obese asthmatic children as the systemic pro-inflammatory status and high level of serum leptin in obese children could mask the local airway inflammation.

Less is known about leptin-mediated molecular mechanisms in human airway inflammation and asthma. However, in recent years, different studies conducted on human bronchial cells [95,96] or mice models of obese asthma [97] have shed some light on this mechanism. Hao et al. [95] and Watanabe et al. [96] analyzed the effect of leptin on human bronchial epithelial cells’ cultures (HBE16) and human lung fibroblasts, respectively (NHLFs). According to those studies, when HBE16 cells line are exposed to IL-13, mucus secretion would be increased by the upregulation of MUC5AC encoding gene transcription, with this process being mediated by leptin. This mechanism seems to be modulated by leptin activation of the JAK_2_-STAT_3_ intracellular signaling pathway. In parallel, when Normal Human Lung Fibroblasts (NHLFs) cells are cultured under high concentrations of leptin, the production of inflammatory cytokines and myofibroblast differentiation and proliferation is enhanced by the interaction of leptin with LEPR, which is widely expressed by fibroblasts. According to the reported studies, NHLFs stimulation resulted in an induction of chemo-attractants production, including eotaxin, IL-8 or monocyte chemoattractant protein-1 (MCP-1). These cytokines would recruit inflammatory cells, induce cells’ degranulation and myofibroblasts differentiation, contributing to asthma exacerbation and epithelial remodeling. With regard to animal models, Chong et al. [97] designed a mice model of obese asthma and after sensitization with intra-parenteral ovalbumin, the animals were sacrificed and bronchoalveolar lavage fluid was collected in order to analyze leptin levels and expression of cellular transcriptional and translational factors. According to their results, leptin levels were higher in Bronchoalveolar lavage (BALF) from obese and asthma-induced animals when compared with non-obese asthmatic and non-asthmatic mice. What is more, when the role of leptin on pulmonary inflammation was analyzed, it was associated to JAK/STAT_3_ upregulation. As can be seen, studies based on cell cultures and mice models are in concordance, constituting a possible explanation for the mechanism involved in airway inflammation and asthma in humans.

Although the role of resistin in asthma pathogenesis is less known, it has been proposed that resistin serum levels and the resistin:adiponectin ratio could be a predictor of asthma risk and lung function in asthma. Ballantyne et al. [98] conducted a cross-sectional observational study, including 96 asthmatic adults and 46 healthy controls. According to their results, plasmatic resistin levels and the resistin:adiponectin ratio were higher in asthmatic patients than in controls, presenting an inverse correlation with respiratory functional parameters: FEV1% and FEV1/FVC (Forced expiratory volume: FEV; Forced vital capacity: FVC). In the case of the resistin:adiponectin ratio, these differences where higher for obese asthmatic patients, as serum adiponectin levels decrease in obese subjects. However, in contrast to adiponectin, resistin levels were not affected by BMI, which agrees with the previously exposed studies, according to which, resistin secretion is mainly induced by high glucose levels and inflammatory mediators [91]. Different studies have analyzed the resistin-mediated mechanisms in obstructive airway diseases, including asthma. In this respect, Fang et al. [99] analyzed the expression of resistin-like molecule β (p bronchial biopsies from allergic patients, and Resistin-like molecule-beta (RELM-β) effect on both human lung fibroblast cell cultures (MRC5)), and mice previously sensitized and exposed against *Aspergillus fumigatus*. Those authors concluded that RELM-β was highly expressed in airway epithelium from asthmatic patients. Its high expression was associated to the increment of TGF-β local production, which seems to promote proliferation of lung fibroblasts and their differentiation to myofibroblasts. This mechanism would increase subepithelial matrix deposition and would contribute to airway fibrosis, which are crucial for obstructive airway diseases’ evolution, such as asthma, since it comprises the reversibility of obstruction. In the same way, Kwak et al. [100] demonstrated that, similar to leptin, resistin increases mucin secretion in human airway epithelial cells through the expression of MUC5AC and MUC5B encoding genes. This mechanism seems to be mediated by NF-Kβ. For that reason, the over-presence of resistin in respiratory epithelium and the over-transcription of MUC encoding genes could be associated to severe asthma, as mucus hypersecretion contributes to airway-obstructive diseases’ pathogenesis [101].

**Figure 1:**
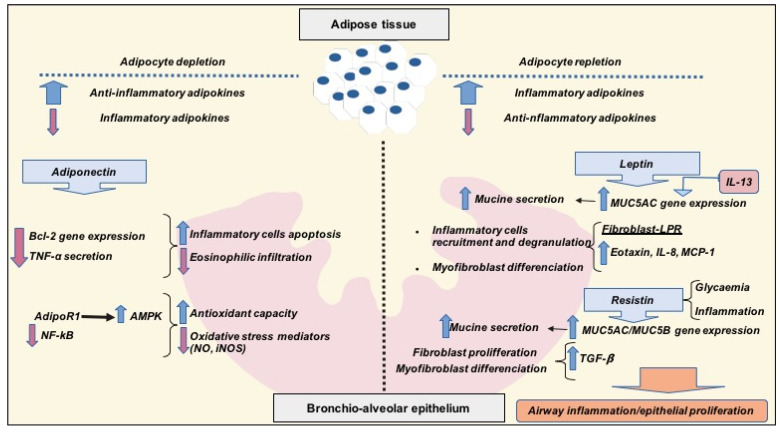
Role of pro-inflammatory and anti-inflammatory adipokines in airway inflammation scheme. Adipocyte depletion is associated with high levels of adiponectin, favoring airway anti-inflammatory status by increasing inflammatory cells’ apoptosis and antioxidant capacity, but decreasing eosinophilic infiltration and reducing the production of oxidative stress mediators. This anti-inflammatory status would be mediated by a downregulation of the inhibitory apoptosis gene Bcl-2, inhibition of TNF-α secretion by airway macrophages and the increment of intracellular AMP-kinase activity through activation of AdipoR1 receptors. A pro-inflammatory cytokine such as leptin is related to obesity and adipocytes’ repletion, whereas resistin levels are increased under high glycaemia and the influence of inflammatory factors. Both leptin and resistin high levels would increase airway mucin secretion through MUC-gene expression, as well as favoring fibroblast proliferation, myofibroblast differentiation and local inflammatory cells’ recruitment through the secretion of pro-inflammatory cytokines (Eotaxin, IL-8, MCP-1 or TGF-β). In this way, high plasmatic levels of leptin and resistin could favor airway inflammation and epithelial remodeling, which are crucial for pulmonary obstructive diseases’ severity and their evolution. Abbreviations: TNF, tumor necrosis factor; NO, nitric oxide; iNOS, inducible nitric oxide synthase isoforms; AmpK, AMP-activated protein kinase; Bcl-2, B-cell lymphoma type 2 gene; NF-kB, nuclear factor kappa-light-chain-enhancer of activated cells; IL, interleukin; MUC5AC Mucin 5AC precursor gene; TGF-β, Tumor growth factor b; MCP-1, Monocyte chemoattractant protein-1.

In summary, both obesity and adipocytes’ repletion have been associated with an imbalance in adipokines’ serum concentration, increasing pro-inflammatory adipokines such as leptin or resistin, and decreasing anti-inflammatory cytokines such as adiponectin. In recent years, this imbalance has been related to different inflammatory-related chronic diseases, including asthma. In the case of asthma and obstructive pulmonary disease, pro-inflammatory cytokines seem to increase the risk of asthma and its severity. More specifically, according to the bibliography reviewed, leptin and resistin would lead to an induction of airway inflammation and epithelial proliferation and fibrosis through fibroblast differentiation stimulation and pro-inflammatory cytokines secretion. On the contrary, caloric restriction and adipocytes’ depletion are associated to high adiponectin levels in serum, which would decrease local airway inflammation and increase local antioxidant capacity. The previously described mechanisms have been summarized in Figure 1.

## 4. Concluding Remarks and Future Directions

Dietary patterns play a main role in the risk and development of many diseases, including asthma. Western lifestyle implies a regular intake of foods containing high amounts of saturated fats and simple sugars that also suppose a reduced intake of complex carbohydrates, unsaturated fats and antioxidant compounds present in plant-origin food. In this frame, the obesity epidemic is highly related with Western/unhealthy dietary patterns and with the alarming and simultaneous growth of inflammatory diseases such as asthma. Related to this, more information about the risk associated with Westernized lifestyles should be managed in order to avoid a detrimental reprogramming of the immune response to be more inflammatory, which can lead to more severe diseases, as occurs when a Western diet is regularly consumed. Dietary management, encouraging the consumption of unsaturated fats rather than foods rich in saturated fats, and promoting the consumption of whole grain-derived foods and plant-origin foods, will provide a diet with anti-inflammatory properties and, probably, with a positive effect on adipose tissue and airway inflammatory processes. Thus, incorporating adequate dietary patterns promoting the Mediterranean diet and insisting on the importance of reducing the intake of sugar-sweetened beverages and highly processed foods into the clinical management of asthma is to be highly recommended.

## References

[1] Christ A., Latz E. (2019). The Western lifestyle has lasting effects on metaflammation. Nat. Rev. Immunol..

[2] Periyalil H.A., Wood L.G., Wright T.A., Karihaloo C., Starkey M.R., Miu A.S., Baines K.J., Hansbro P.M., Gibson P.G. (2018). Obese asthmatics are characterized by altered adipose tissue macrophage activation. Clin. Exp. Allergy.

[3] Sin D.D., Sutherland E.R. (2008). Obesity and the lung: 4. Obesity and asthma. Thorax.

[4] Rush E.C., Yan M.R. (2017). Evolution not Revolution: Nutrition and Obesity. Nutrients.

[5] Braback L., Hjern A., Rasmussen F. (2004). Trends in asthma, allergic rhinitis and eczema among Swedish conscripts from farming and non-farming environments. A nationwide study over three decades. Clin. Exp. Allergy.

[6] Haahtela T., Lindholm H., Bjorksten F., Koskenvuo K., Laitinen L.A. (1990). Prevalence of asthma in Finnish young men. BMJ.

[7] Eder W., Ege M.J., von Mutius E. (2006). The asthma epidemic. N. Engl. J. Med..

[8] García-Marcos L., Quiros A.B., Hernández G.G., Guillen-Grima F., Díaz C.G., Ureña I.C., Peña A.A., Monge R.B., Suarez-Varela M.M., Varela A.L. (2004). Stabilization of asthma prevalence among adolescents and increase among schoolchildren (ISAAC phases I and III) in Spain. Allergy.

[9] Asher M.I., Montefort S., Bjorksten B., Lai C.K., Strachan D.P., Weiland S.K., Williams H. (2006). Worldwide time trends in the prevalence of symptoms of asthma, allergic rhinoconjunctivitis, and eczema in childhood: ISAAC Phases One and Three repeat multicountry cross-sectional surveys. Lancet.

[10] Hales C.M., Fryar C.D., Carroll M.D., Freedman D.S., Ogden C.L. (2018). Trends in Obesity and Severe Obesity Prevalence in US Youth and Adults by Sex and Age, 2007–2008 to 2015–2016. JAMA.

[11] Ogden C.L., Fryar C.D., Hales C.M., Carroll M.D., Aoki Y., Freedman D.S. (2018). Differences in Obesity Prevalence by Demographics and Urbanization in US Children and Adolescents, 2013–2016. JAMA.

[12] Hallstrand T.S., Fischer M.E., Wurfel M.M., Afari N., Buchwald D., Goldberg J. (2005). Genetic pleiotropy between asthma and obesity in a community-based sample of twins. J. Allergy Clin. Immunol..

[13] Thomsen S.F., Ulrik C.S., Kyvik K.O., Sørensen T.I., Posthyma D., Skadhauge L.R., Steffensen I., Backer V. (2007). Association between obesity and asthma in a twin cohort. Allergy.

[14] Garcia-Marcos L., Canflanca I.M., Garrido J.B., Varela A.L., Garcia-Hernandez G., Guillen-Grima F., Gonzalez-Diaz C., Carbajal-Ureña I., Arnedo-Peña A., Busquets-Monge R.M. (2007). Relationship of asthma and rhinoconjunctivitis with obesity, exercise and Mediterranean diet in Spanish schoolchildren. Thorax.

[15] Garcia-Marcos L., Castro-Rodriguez J.A., Weinmayer G., Panagiotakos D.B., Priftis K.N., Nagel G. (2013). Influence of Mediterranean diet on asthma in children: A systematic review and meta-analysis. Pediatr. Allergy Immunol..

[16] Ellwood P., Asher M.I., Garcia-Marcos L., Williams H., Keil U., Robertson C., Nagel G. (2013). Do fast foods cause asthma, rhinoconjunctivitis and eczema? Global findings from the International Study of Asthma and Allergies in Childhood (ISAAC) phase three. Thorax.

[17] Papadopoulou A., Panagiotakos D.B., Hatziagorou E., Antonogeorgos G., Matziou V.N., Tsanakas J.N., Gratziou C., Tsabouri S., Priftis K.N. (2015). Antioxidant foods consumption and childhood asthma and other allergic diseases: The Greek cohorts of the ISAAC II survey. Allergol. Immunopathol. (Madr).

[18] Sahiner U.M., Birben E., Erzurum S., Sackesen C., Kalayci O. (2018). Oxidative stress in asthma: Part of the puzzle. Pediatr. Allergy Immunol..

[19] Wall C.R., Stewart A.W., Hancox R.J., Murphy R., Braithwaite I., Beasley R., Mitchell E.A. (2018). Association between frequency counsumption of fruit, vegetables, nuts and pulses and BMI: Alayses of the International study of Asthma and Allergies in Childhood. Nutrients.

[20] Amati F., Hassounah S., Swaka A. (2019). The Impact of Mediterranean Dietary Patterns During Pregnancy on Maternal and Offspring Health. Nutrients.

[21] Forno E., Young O.M., Kumar R., Simhan H., Celedon J.C. (2014). Maternal obesity in pregnancy, gestional weight gain and risk of childhood asthma. Pediatrics.

[22] Tie H.T., Xia Y.Y., Zeng Y.S., Zhang Y., Dai C.L., Guo J.J., Zhao Y. (2014). Risk of childhood overweight or obesity associated with excessive weight gain during pregnancy: A meta-analysis. Arch. Gynecol. Obstet..

[23] Nagel G., Weinmayr G., Kleiner A., García-Marcos L., Strachan D.P. (2010). Effect of diet on asthma and allergic sensitization in the International Studies on Allergies and Asthma in Childhood (ISAAC) Phase Two. Thorax.

[24] Alwarith J., Kahleova H., Crosby L., Brooks A., Brandon L., Levin S.M., Barnard N.D. (2020). The role of nutrition in asthma prevention and treatment. Nutr. Rev..

[25] Schulze M.B., Martínez-González M.A., Fung T.T., Lichtenstein A.H., Forouhi N.H. (2018). Food based dietary patterns and chronic disease prevention. BMJ.

[26] Younas H., Vieira M., Gu C., Lee R., Shin M.K., Berger S., Loube J., Nelson A., Bevans-Fonti S., Zhong Q. (2019). Caloric restriction prevents the development of airway hyperresponsiveness in mice on a high fat diet. Sci. Rep..

[27] Castro Rodríguez J.A., Garcia Marcos L. (2017). What is the effect of a Mediterranean diet on allergies and asthma in children?. Front. Pediatr..

[28] Brandhorst S., Longo V.D. (2019). Dietary restrictions and nutrition in the prevention and treatment of cardiovascular disease. Circ. Res..

[29] López-Guarnido O., Urquiza N., Saiz M., Lozano D., Rodrigo L., Pascual M., Lorente J.A., Alvarez-Cubero M.J., Rivas A. (2018). Bioactive compounds of the Mediterranean diet and prostate cancer. Aging Male.

[30] Brigham E.P., Kolahdooz F., Hansel N., Breysse P.N., Davis M., Sharma S., Matsui E.C., Diette G., McCormack M.C. (2015). Association between Western diet pattern and adult asthma: A focused review. Ann. Allergy Asthma Immunol..

[31] Park Y., Subar A.F., Hollenbeck A., Schatzkin A. (2011). Dietary fiber intake and mortality in the NIH-AARP diet and health study. Arch. Intern. Med..

[32] Calder P.C. (2013). Omega-3 polyunsaturated fatty acids and inflammatory processes: Nutrition or pharmacology?. Br. J. Clin. Pharmacol..

[33] Andersen C.J., Fernandez M.L. (2013). Dietary strategies to reduce metabolic syndrome. Rev. Endocr. Metab. Dis..

[34] Sarin H.V., Lee H., Jauhiainen M., Joensuu A., Borodulin K., Männistö S., Jin Z., Terwilliger J.D., Isola V., Ahtiainen P. (2019). Substantial fat mass loss reduces low-grade inflammation and induces positive alteration in cardiometabolic factors in normal-weight individuals. Nat. Sci. Rep..

[35] Duwaerts C.C., Maher J.J. (2019). Macronutrients and the adipose-liver axis in obesity and fatty liver. Cell. Mol. Gastroenterol. Hepatol..

[36] Ellulu M.S., Patimah I., Khazáai H., Rahmat A., Abed Y. (2017). Obesity and inflammation: The linking mechanism and the complications. Arch. Med. Sci..

[37] Maggini S., Pierre A., Calder P.C. (2018). Inmune function and micronutrient requirements change over the life course. Nutrients.

[38] Jensen M.K., Koh-Banerjee P., Franz M., Sampson L., Gronbaek M., Rimm E.B. (2006). Whole grains, bran and germ in relation to homocysteine and markers of glycemic control, lipids and inflammation. Am. J. Clin. Nutr..

[39] Boulangé C.L., Neves A.L., Chilloux J., Nicholson J.K., Dumas M.E. (2016). Impact of the gut microbiota, obesity, and metabolic disease. Genome Med..

[40] DeChristopher L.R., Tucker K.L. (2018). Excess of free fructose, high-fructose corn syrup and adult asthma: The Framingham Offspring Cohort. Br. J. Nutr..

[41] Botchlett R., Chaodong W. (2018). Diet composition for the management of obesity and obesity-related disorders. J. Diabetes Mellit. Metab. Syndr..

[42] Wendel S.G., Baffi C., Holguin F. (2014). Fatty acids, inflammation and asthma. J. Allergy Clin. Inmunol..

[43] Sureda A., Bibiloni M.M., Julibert A., Bouzas C., Argelich E., Llompart I., Pons A., Tur J.A. (2018). Adherence to the Mediterranean Diet and inflammatory markers. Nutrients.

[44] Wood L.G., Li Q., Scott H.A., Rutting S., Berthon B.S., Gibson P.G., Hansbro P.M., Williams E., Horvat J., Simpson J.L. (2019). Saturated fatty acids, obesity, and the nucleotide oligomerization domain-like receptor protein 3 (NLRP3) inflammasome in asthmatic patients. J. Allergy Clin. Inmunol..

[45] Wood L.G., Shivappa N., Berthon B.S., Gibson P.G., Hebert J.R. (2015). Dietary inflammatory index is related to asthma risk, lung function and systemic inflammation in asthma. Clin. Exp. Allergy.

[46] Kim J.H., Elwood P.E., Asher M.I. (2009). Diet and asthma: Looking back, moving forward. Respir. Res..

[47] Dandona P., Ghanim H., Chaudhuri A., Dhindsa S., Kim S.S. (2010). Macronutrient intake induces oxidative and inflammatory stress: Potential relevance to atherosclerosis and insulin resistance. Exp. Mol. Med..

[48] Sofi F., Macchi C., Abbate R., Gensini G.F., Casini A. (2013). Mediterranean diet and health. Biofactors.

[49] Te Morenga L., Montez J.M. (2017). Health effects of saturated and trans-fatty acid intake in children and adolescents: Systematic review and meta-analysis. PLoS ONE.

[50] Sievert K., Lawrence M., Naika A., Baker P. (2019). Processed foods and nutrition transition in the Pacific: Regional trends, patterns and food system drivers. Nutrients.

[51] WHO (2019). Essential Nutrition Actions: Mainstreaming Nutrition throughout the Life-Course.

[52] Li Z., Rava M., Bédard A., Dumas O., Garcia-Aymerich J., Leynaert B., Pison C., Le Moual N., Romieu I., Siroux V. (2017). Cured meat intake is associated with worsening asthma symptoms. Thorax.

[53] Brathwaite N., Fraser H.S., Modeste N., Broome H., King R. (2003). Obesity, diabetes, hypertension, and vegetarian status among Seventh-Day Adventists in Barbados: Preliminary results. Ethn. Dis..

[54] Andrianasolo R., Kesse-Guyot E., Moufidath A., Hercberg S., Galan P., Varraso R. (2018). Association between cured meat intake and asthma symptoms. Eur. Respir. J..

[55] Statovci D., Aguilera M., MacSharry J., Melgar S. (2017). The impact of Western diet and nutrients on the microbiota and immune response at mucosal interfaces. Front. Immunol..

[56] Pfeffer P.E., Hawrylowicz C.M. (2018). Vitamin D in asthma. Mechanisms of action and considerations for clinical trials. CHEST.

[57] Scott H.A., Gibson P.G., Garg M.L., Wood L.G. (2011). Airway inflammation is augmented by obesity and fatty acids in asthma. Eur. Respir. J..

[58] Umetsu D.T. (2017). Mechanisms by which obesity impacts upon asthma. Thorax.

[59] Pérez-Pérez A., Vilariño-García T., Fernández-Riejos P., Martín-González J., Segura-Egea J.J., Sánchez-Margalet V. (2017). Role of leptin as a link between metabolism and the immune system. Cytokine Growth Factor Rev..

[60] Scott H.A., Gibson P.G., Garg M.L., Pretto J.J., Morgan P.J., Callister R., Wood L.G. (2012). Relationship between body composition, inflammation and lung function in overweight and obese asthma. Respir. Res..

[61] Dixon A.A., Poynter M.E. (2016). Mechanisms of asthma in obesity: Pleiotropic aspects of obesity produce distinct asthma phenotypes. Am. J. Respir. Cell Mol. Biol..

[62] Frati F., Salvatori C., Incorvaia C., Bellucci A., Di Cara G., Marcucci F., Esposito S. (2019). The role of the microbiome in asthma: The gut-lung axis. Int. J. Mol. Sci..

[63] Samir P., Malireddi S., Kanneganti T.D. (2018). Food for training- Western diet and inflammatory memory. Cell Metab..

[64] Trayhurn P., Wood I.S. (2004). Adipokines: Inflammation and the pleiotropic role of white adipose tissue. Br. J. Nutr..

[65] Mancuso P. (2016). The role of adipokines in chronic inflammation. Immunotargets Ther..

[66] Ambroszkiewicz J., Chełchowska M., Rowicka G., Klemarczyk W., Strucińska M., Gajewska J. (2018). Anti-Inflammatory and Pro-Inflammatory Adipokine Profiles in Children on vegetarian and Omnivorous Diets. Nutrients.

[67] Peake P.W., Kriketos A.D., Campbell L.V., Shen Y., Charlesworth J.A. (2005). The metabolism of isoforms of human adiponectin: Studies in human subjects and in experimental animals. Eur. J. Endocrinol..

[68] Salehi-Abargouei A., Izadi V., Azadbakht L. (2015). The effect of low calorie diet on adiponectin concentration: A systematic review and meta-analysis. Horm. Metab. Res..

[69] Zhu N., Pankow J.S., Ballantyne C.M., Couper D., Hoogeveen R.C., Pereira M., Duncan B.B., Schmidt M.I. (2010). High-molecular-weight adiponectin and the risk of type 2 diabetes in the ARIC study. J. Clin. Endocrinol. Metab..

[70] Iwata M., Hara K., Kamura Y., Honoki H., Fujisaka S., Ishiki M., Usui I., Yagi K., Fukushima Y., Takano A. (2018). Ratio of low molecular weight serum adiponectin to the total adiponectin value is associated with type 2 diabetes through its relation to increasing insulin resistance. PLoS ONE.

[71] Liu Z., Liang S., Que S., Zhou L., Zheng S., Mardinoglu A. (2018). Meta-analysis of adiponectin as a biomarker for the detection of metabolic syndrome. Front. Physiol..

[72] Nigro E., Daniele A., Scudiero O., Ludovica-Monaco M., Roviezzo F., D’Agostino B., Mazzarella G., Bianco A. (2015). Adiponectin in asthma: Implications for phenotyping. Curr. Protein Pept. Sci..

[73] Denzel M.S., Scimia M.C., Zumstein P.M., Walsh K., RuizLozano P., Ranscht B. (2010). T-cadherin is critical for adiponectin mediated cardioprotection in mice. J. Clin. Investig..

[74] Parker-Duffen J.L., Nakamura K., Silver M., Kikuchi R., Tigges U., Yoshida S., Denzel M.S., Ranscht B., Walsh K. (2013). T-cadherin is essential for adiponectin-mediated revascularization. J. Biol. Chem..

[75] Kalisz M., Baranowska B., Wolińska-Witort E., Mączewski M., Mackiewicz U., Tułacz D., Gora M., Martynska L., Bik W. (2015). Total and high molecular weight adiponectin levels in the rat model of post-myocardial infarction heart failure. J. Physiol. Pharmacol..

[76] Jaswal S., Saini V., Kaur J., Gupta S., Kaur H., Garg K. (2018). Association of Adiponectin with Lung Function Impairment and Disease Severity in Chronic Obstructive Pulmonary Disease. Int. J. Appl. Basic. Med. Res..

[77] Otero M., Lago R., Gomez R., Lago F., Dieguez C., Gómez-Reino J.J., Gualillo O. (2006). Changes in plasma levels of fat-derived hormones adiponectin, leptin, resistin and visfatin in patients with rheumatoid arthritis. Ann. Rheum. Dis..

[78] Kirdar S., Serter M., Ceylan E., Sener A.G., Kavak T., Karadağ F. (2009). Adiponectin as a biomarker of systemic inflammatory response in smoker patients with stable and exacerbation phases of chronic obstructive pulmonary disease. Scand. J. Clin. Lab. Investig..

[79] Sood A., Shore S.A. (2013). Adiponectin, leptin, and resistin in asthma: Basic mechanisms through population studies. J. Allergy.

[80] Ma C., Wang Y., Xue M. (2019). Correlations of severity of asthma in children with body mass index, adiponectin and leptin. J. Clin. Lab. Anal..

[81] Wahab A., Maarafiya M.M., Ashraf Soliman A., Noura B.M., Younes N.B.M., Chandra P. (2013). Serum Leptin and Adiponectin Levels in Obese and Nonobese Asthmatic School Children in relation to Asthma Control. J. Allergy.

[82] Yuksel H., Sogut A., Yilmaz O., Onur E., Dinc G. (2012). Role of adipokines and hormones of obesity in childhood asthma. Allergy Asthma Immunol. Res..

[83] Zhu L., Chen X., Chong L., Kong L., Wen S., Zhang H., Zhang W., Li C. (2019). Adiponectin alleviates exacerbation of airway inflammation and oxidative stress in obesity-related asthma mice partly through AMPK signaling pathway. Int. Immunopharmacol..

[84] Friedman J.M., Halaas J.L. (1998). Leptin and the regulation of body weight in mammals. Nature.

[85] Heisler L.K., Lam D.D. (2017). An appetite for life: Brain regulation of hunger and satiety. Curr. Opin. Pharmacol..

[86] Otero M., Lago R., Gomez R., Dieguez C., Lago F., Gomez-Reino J., Gualillo O. (2006). Towards a pro-inflammatory and immunomodulatory emerging role of leptin. Rheumatology.

[87] Frühbeck G. (2006). Intracellular signalling pathways activated by leptin. Biochem. J..

[88] Abella V., Scotece M., Conde J., Pino J., Gonzalez-Gay M.A., Gomez-Reino J.J., Mera A., Lago F., Gomez R., Gualillo O. (2017). Leptin in the interplay of inflammation, metabolism and immune system disorders. Nat. Rev. Rheumatol..

[89] Schwartz D.R., Lazar M.A. (2011). Human resistin: Found in translation from mouse to man. Trends Endocrinol. Metab..

[90] Lee S., Lee H.C., Kwon Y.W., Lee S.E., Cho Y., Kim J., Lee S., Kim J.Y., Lee J., Yang H.M. (2014). Adenylyl cyclase-associated protein 1 is a receptor for human resistin and mediates inflammatory actions of human monocytes. Cell Metab..

[91] Park H.K., Kwak M.K., Kim H.J., Ahima R.S. (2017). Linking resistin, inflammation, and cardiometabolic diseases. Korean J. Intern. Med..

[92] Sood A., Ford E.S., Camargo C.A. (2006). Association between leptin and asthma in adults. Thorax.

[93] Guler N., Kirerleri E., Ones U., Tamay Z., Salmayenli N., Darendeliler F. (2004). Leptin: Does it have any role in childhood asthma?. J. Allergy Clin. Immunol..

[94] Bodini A., Tenero L., Sandri M., Maffeis C., Piazza M., Zanoni L., Peroni D., Boner A., Piacentini G. (2017). Serum and exhaled breath condensate leptin levels in asthmatic and obesity children: A pilot study. J. Breath Res..

[95] Hao W., Wang J., Zhang Y., Wang Y., Sun L., Han W. (2017). Leptin positively regulates MUC5AC production and secretion induced by interleukin-13 in human bronchial epithelial cells. Biochem Biophys. Res. Commun..

[96] Watanabe K., Suzukawa M., Arakawa S., Kobayashi K., Igarashi S., Tashimo H., Nagai H., Tohma S., Nagase T., Ohta K. (2019). Leptin enhances cytokine/chemokine production by normal lung fibroblasts by binding to leptin receptor. Allergol. Int..

[97] Chong L., Liu L., Zhu L., Li H., Shao Y., Zhang H., Yu G. (2019). Expression Levels of Predominant Adipokines and Activations of STAT3, STAT6 in an Experimental Mice Model of Obese Asthma. Iran. J. Allergy Asthma Immunol..

[98] Ballantyne D., Scott H., MacDonald-Wicks L., Gibson P.G., Wood L.G. (2016). Resistin is a predictor of asthma risk and resistin: Adiponectin ratio is a negative predictor of lung function in asthma. Clin. Exp. Allergy.

[99] Fang C.L., Yin L.J., Sharma S., Kierstein S., Wu H.F., Eid G., Haczku A., Corrigan C.J., Ying S. (2015). Resistin-like molecule-β (RELM-β) targets airways fibroblasts to effect remodelling in asthma: From mouse to man. Clin. Exp. Allergy.

[100] Kwak S., Kim Y.D., Na H.G., Bae C.H., Song S.Y., Choi Y.S. (2018). Resistin upregulates MUC5AC/B mucin gene expression in human airway epithelial cells. Biochem. Biophys. Res. Commun..

[101] Lachowicz-Scroggins M.E., Yuan S., Kerr S.C., Dunican E.M., Yu M., Carrington S.D., Fahy J.V. (2016). Abnormalities in MUC5AC and MUC5B protein in airway mucus in asthma. Am. J. Respir. Crit. Care Med..

